# A Text Messaging Intervention (StayWell at Home) to Counteract Depression and Anxiety During COVID-19 Social Distancing: Pre-Post Study

**DOI:** 10.2196/25298

**Published:** 2021-11-01

**Authors:** Adrian Aguilera, Rosa Hernandez-Ramos, Alein Y Haro-Ramos, Claire Elizabeth Boone, Tiffany Christina Luo, Jing Xu, Bibhas Chakraborty, Chris Karr, Sabrina Darrow, Caroline Astrid Figueroa

**Affiliations:** 1 School of Social Welfare University of California, Berkeley Berkeley, CA United States; 2 Department of Psychiatry and Behavioral Sciences University of California San Francisco San Francisco, CA United States; 3 School of Public Health University of California, Berkeley Berkeley, CA United States; 4 Data Science Programme, Division of Science and Technology Beijing Normal University-Hong Kong Baptist University United International College Zhuhai China; 5 Center for Quantitative Medicine Duke National University of Singapore Singapore Singapore; 6 Department of Biostatistics and Bioinformatics Duke University Durham, NC United States; 7 Department of Statistics and Applied Probability National University of Singapore Singapore Singapore; 8 Audacious Software Chicago, IL United States

**Keywords:** mobile health, COVID-19, text messaging, cognitive behavioral therapy, anxiety, depression, microrandomized trials, mHealth, intervention, mental health, SMS

## Abstract

**Background:**

Social distancing and stay-at-home orders are critical interventions to slow down person-to-person transmission of COVID-19. While these societal changes help contain the pandemic, they also have unintended negative consequences, including anxiety and depression. We developed StayWell, a daily skills-based SMS text messaging program, to mitigate COVID-19–related depression and anxiety symptoms among people who speak English and Spanish in the United States.

**Objective:**

This paper describes the changes in StayWell participants’ anxiety and depression levels after 60 days of exposure to skills-based SMS text messages.

**Methods:**

We used self-administered, empirically supported web-based questionnaires to assess the demographic and clinical characteristics of StayWell participants. Anxiety and depression were measured using the 2-item Generalized Anxiety Disorder (GAD-2) scale and the 8-item Patient Health Questionnaire-8 (PHQ-8) scale at baseline and 60-day timepoints. We used 2-tailed paired *t* tests to detect changes in PHQ-8 and GAD-2 scores from baseline to follow-up measured 60 days later.

**Results:**

The analytic sample includes 193 participants who completed both the baseline and 60-day exit questionnaires. At the 60-day time point, there were significant reductions in both PHQ-8 and GAD-2 scores from baseline. We found an average reduction of –1.72 (95% CI –2.35 to –1.09) in PHQ-8 scores and –0.48 (95% CI –0.71 to –0.25) in GAD-2 scores. These improvements translated to an 18.5% and 17.2% reduction in mean PHQ-8 and GAD-2 scores, respectively.

**Conclusions:**

StayWell is an accessible, low-intensity population-level mental health intervention. Participation in StayWell focused on COVID-19 mental health coping skills and was related to improved depression and anxiety symptoms. In addition to improvements in outcomes, we found high levels of engagement during the 60-day intervention period. Text messaging interventions could serve as an important public health tool for disseminating strategies to manage mental health.

**Trial Registration:**

ClinicalTrials.gov NCT04473599; https://clinicaltrials.gov/ct2/show/NCT04473599

**International Registered Report Identifier (IRRID):**

RR2-10.2196/23592

## Introduction

The COVID-19 pandemic is a significant public health crisis that has caused devastating physical illness and concurrent mental health challenges [1]. Public health measures, including stay-at-home orders and the closure of nonessential businesses, have been necessary to reduce transmission but have also disrupted social life by limiting social activities and physical interactions with one’s networks [2,3].

Societal changes to contain the COVID-19 pandemic have caused significant psychological distress worldwide in people of various backgrounds [1,4-6]. Studies show lowered psychological well-being and increased depressive and anxiety symptoms among the general public compared to prepandemic rates [1]. In the United States, the risk of depression among adults increased 3-fold during the COVID-19 pandemic compared to before the pandemic [7]. Stressors associated with social distancing and loss of usual routines, including infection fear, financial insecurity, frustration, and a sense of isolation, had negative psychological impacts, including increased depression and anxiety symptoms [4]. These stressors also increased insomnia [8], decreased physical activity [9], and increased alcohol and substance use [10] in diverse global samples.

While the prevalence of anxiety and depression has increased in the general population, certain groups are at a higher risk of mental health disorders. Individuals with a greater risk for depression during the pandemic include those from lower socioeconomic backgrounds with insufficient economic resources, inadequate social support, and greater exposure to social stressors, such as pandemic-related job loss [7]. Furthermore, the pandemic has disproportionately affected the health of already at-risk individuals such as those from low-income backgrounds, communities of color, and non–English-speaking groups [11].

Text messaging is a promising tool to deliver interventions that address the detrimental mental health effects of the COVID-19 pandemic [12-15], especially for underserved populations [16,17]. Texting, often viewed as the “workhorse” of digital and mobile health, is a widely used communication strategy that has been leveraged to deliver mental health interventions by relaying health information, skills-based messages, and self-monitoring messages to participants [16]. Text messaging interventions can help fill the gap between the need for and availability of mental health services, including behavioral health appointments, a gap that worsened during the pandemic [18]. Because 85% of all US adults and 76% of lower income adults own a smartphone, texting interventions have the capacity to reach a large and diverse group of people [19]. Expanding the reach of mental health programs is especially crucial since depression and anxiety symptoms have increased in the general population and intensified among those with existing mental health disorders and vulnerable communities.

Incorporating cognitive behavioral therapy (CBT) and skills-based text messaging interventions have proved feasible and acceptable among a diverse group of patients with affective disorders [20]. A significant body of research has established the effectiveness of CBT as an evidence-based, first-line treatment for mental health conditions such as depression and anxiety [21]. CBT has been implemented in diverse populations, including communities of color and individuals of lower socioeconomic status. Additionally, CBT is a focused, directive, and structured form of psychotherapy, making it well suited for delivery via digital platforms such as text messaging. Electronically delivered CBT is at least as effective as face-to-face CBT at reducing mental health symptoms [22].

In April 2020, we initiated the StayWell at Home intervention (ClinicalTrials.gov, NCT04473599), a 60-day skills-based daily text messaging program in accordance with principles of CBT [23]. This paper assesses the effects of StayWell on symptoms of depression and anxiety in a broad adult population living in the United States during the COVID-19 pandemic. Texts were based on two core components of CBT: behavioral activation (BA) and psychoeducation. BA aims to help people engage in enjoyable activities, reduce reliance on unhealthy coping mechanisms, and decrease avoidance of anxiety-provoking situations. By directing individuals to pleasurable and meaningful activities, BA can improve mood and decrease loneliness and isolation related to the pandemic. Providing psychoeducation around thoughts, feelings, and behaviors is also an important part of CBT. Messages focused on promoting adaptive cognitive approaches to pandemic-related stress and encouraged BA within the limits of social distancing. Maladaptive thoughts and behaviors can be identified and replaced to reduce the frequency and intensity of negative emotions. Further, information and reminders related to self-care, sleep, physical activity, and mindfulness may promote positive health behavior change and have beneficial effects on individuals’ psychological well-being. We hypothesize that participants in the intervention will report fewer depression and anxiety symptoms at the end of the intervention.

## Methods

### StayWell Trial Design

StayWell is a fully remote trial and has various designs: (1) a pre-post comparison, in which we assessed depression and anxiety symptoms for all patients before and after the intervention and (2) a randomized controlled trial with two groups: Uniform Random (UR) messaging and a Reinforcement Learning (RL) messaging. Owing to a coding error in the algorithm, all participants received messages randomly (UR condition). Therefore, we altered the study’s main aims by focusing on the pre-post effects of participating in StayWell on depression and anxiety symptoms. The institutional review board of the University of California (UC) Berkeley reviewed and approved all study procedures.

Participants were enrolled in the StayWell trial using the HealthySMS platform—an automated text messaging platform developed by the authors [20,24]. HealthySMS has been successfully used with various low-income English- and Spanish-speaking populations to send automated text messages and manage participant responses using a secure, Health Insurance Portability and Accountability Act–compliant platform [20]. Participants received 2 messages daily for 60 days: 1 skills-based message and 1 message inquiring about their mood. The skills-based messages included tips on how to deal with worry and stress brought on by the COVID-19 pandemic. Half of these messages were based on BA strategies, and half were based on skill-based strategies. We developed the messages to highlight evidence-based practices used in depression and anxiety interventions that promote behavior change. Templates for these messages were developed from previous work conducted by authors SMD and AA [16]. The study team edited the original messages to fit the COVID-19 context and improve readability. The team also translated and culturally adapted messages in Spanish to expand the reach of the intervention. Messages were sent daily at a random time between 9 AM and 6 PM. Three hours following the delivery of the skills-based messages, participants were sent a message inquiring about their mood on a scale of 1-9, with 9 being the best mood.

### UR Message Arm

Participants received messages uniform randomly (ie, a microrandomized trial design [16]), where every day during the study treatment allocation was characterized by a full factorial design with two factors: skills-based messages (M) and the time frame (T) when the message was sent. M has 2 levels (BA and skill-based), and T has 3 levels (9 AM-12 PM, 12 PM-3 PM, and 3 PM-6 PM). Participants received 1 daily skills-based message and 1 daily mood message that did not vary.

### Data Collection

**Figure 1:**
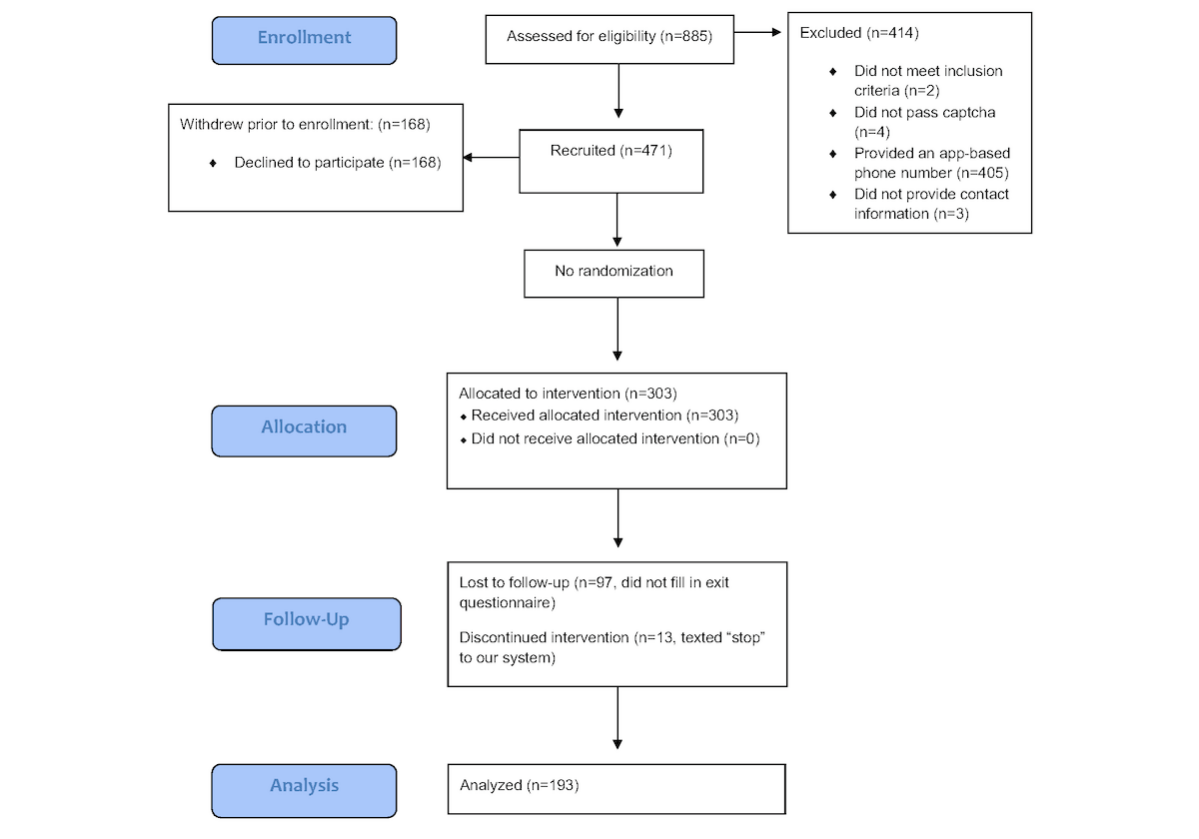
CONSORT (Consolidated Standards of Reporting Trials) flow diagram of the data collection process.

Adult Spanish and English speakers aged 18 years and older who had a mobile phone were recruited via web-based media advertisements on Facebook, Craigslist, and university websites (UC Berkeley and UC San Francisco) to participate. Data were collected through a web-based Qualtrics survey and HealthySMS. Participants were excluded if they used a web-based text messaging app or were outside of the United States. Web-based text messaging apps are more prone to scams on the internet and facilitate the creation of multiple phone numbers for 1 participant. The study lasted from April to December 2020. The CONSORT (Consolidated Standards for Reporting Trials) flow diagram for data collection is shown in Figure 1.

#### Recruitment

We designed web-based advertisements to target vulnerable populations, including low-income groups and people of color, who are disproportionately impacted by the COVID-19 pandemic in the United States. Using a user-centered design, we created 16 user personas. A persona is a fictional characterization of a user, which includes specific characteristics and demographics found in the target population [25]. The personas informed the title, picture, and reason for participating in the study used on each advertisement and the characteristics used for detailed targeted advertising, which is available on Facebook advertisements.

#### Enrollment

To prevent web-based scams and fraud, interested subjects were sent to a Qualtrics survey to assess eligibility criteria and human identity through a built-in *captcha*. Eligible participants were then sent a unique weblink to a baseline assessment. Using a different Qualtrics survey, participants consented and answered demographic questions and other measures of interest. Upon completion, participants were enrolled in an automated text messaging intervention for 60 days. On day 61, participants were sent an exit assessment where they were asked the measures of interests originally asked at baseline. Participants were paid US $20 at the end of the study for completing study questionnaires. 

### Outcome Measures

Our primary outcomes, including the 8-item Patient Health Questionnaire 8 [26] and the 2-item Generalized Anxiety Disorder 2 [27] (GAD-2) scale, were collected through a Qualtrics survey at pre- and postintervention.

Secondarily, we were interested in assessing engagement in the intervention by measuring response rates to mood rating messages and calculating how many participants stopped the text messaging.

### Hypotheses

We will conduct a pre-post comparison among all participants. The depression score measured using the 8-item Patient Health Questionnaire (PHQ-8) and the anxiety score measured using the 2-item Generalized Anxiety Disorder (GAD-2) scale will be improved over the 60-day study.

### Power Analysis

The sample size calculation was performed in a previous protocol paper [23], which includes 2 aims. This paper only considers the primary aim. At a medium standardized effect size (ie, Cohen *d*=0.5), a sample size of 64 participants is required to detect an improvement of either the depression or anxiety score from baseline to 60-day at 80% power and 5% level of significance. The sample size of this study was 193 participants, which is based on the secondary aim. The secondary outcome focuses on the proximal effect of daily improvement on the mood rating. However, the analysis will be presented in a separate manuscript.

### Statistical Analysis

#### Main Analyses

To detect the change in depression (PHQ-8) and anxiety (GAD-2) scores from baseline to follow-up measured 60 days later, we used 2-tailed paired *t* tests. The normality assumptions for the change in each score are validated by their corresponding histogram plots, which are relatively symmetric. The goodness of fit to normal distribution was validated using the Anderson-Darling test, while skewness for normality was validated using the Shapiro-Wilk skewness test [28].

#### Exploratory Analyses

We used simple linear regression analysis to model the change in PHQ-8 and GAD-2 scores as a function of participants’ response rates (ie, the proportion of mood messages answered) to determine whether the improvements in depression and anxiety are predicted by engagement with the intervention. Furthermore, to determine whether any other covariates predict the effects of the StayWell intervention, we used multivariable linear regression analysis to model the change in PHQ-8 and GAD-2 scores (ie, scores at follow-up minus scores at baseline) as a function of demographic predictors, response rates, self-rated health, and the change in COVID-19 weekly rolling average case rates in each participants’ county of residence. Demographic predictors include education (at least high school, some college, college, or graduate degree), age, gender (female, male, or other), language (English or Spanish), and employment (full-time, part-time, unemployed, or other). Self-rated health includes poor/fair, good, very good, and excellent. The weekly rolling average of daily new confirmed cases per 100,000 population is calculated for the day in which participants enroll and exit the program by averaging the values of that day, 3 days prior, and 3 days thereafter. We then used the change in COVID-19 case rates at 60-day follow-up from the baseline date for each participant.

## Results

### Results Overview

A total of 303 people entered the study and received text messages. Of these, 12 were recruited via ClinicalTrials.gov, 75 via Craigslist, 184 via Facebook, and 32 by texting the StayWell phone number. We show the distribution of baseline characteristics in Table 1. Most baseline respondents were female (76.0%) and spoke English (88.4%). While almost half of the respondents identified as White or Caucasian (47.9%), the sample was relatively diverse with 20.5% Latinx, 13.2% Asian or Pacific Islander, 11.5% multiethnic, and 6.6% Black or African American participants. Of the baseline participants, 193 also completed an exit questionnaire and were included in the main analysis.

**Table 1:** Baseline demographic and clinical characteristics (N=303).

Characteristics		Value
Age (years), mean (SD)		33.3 (11.0)
Females, n (%)		230 (76.0)
**Language, n (%)**		
	English	268 (88.4)
	Spanish	35 (11.3)
**Employment, n (%)**		
	Full-time (greater than or equal to 35 hours/week)	137 (45.2)
	Part-time (less than 35 hours)	61 (20.1)
	Homemaker	28 (9.2)
	Unemployed	50 (16.5)
	Disabled/on disability	12 (4.0)
	Retired	1 (0.3)
	Other	14 (4.6)
**Race/ethnicity, n (%)**		
	Asian or Pacific Islander	40 (13.2)
	Black or African American	20 (6.6)
	White or Caucasian	145 (47.9)
	Latino(a) or Hispanic	62 (20.5)
	Multiethnic	35 (11.5)
	Unknown	1 (0.3)
**Education, n (%)**		
	Between 6th and 8th grade	1 (0.3)
	Some high school	10 (3.3)
	High school graduate	29 (9.6)
	Some college or technical school	94 (31.0)
	College graduate	103 (34.0)
	Graduate degree	66 (21.8)
**Paying for basics (eg, food, housing, medical care, and heating) is, n (%)**		
	Very hard	119 (39.3)
	Sometimes hard	137 (45.2)
	Not hard at all	47 (15.5)
**Self-reported health, n (%)**		
	Excellent	8 (2.6)
	Very good	41 (13.5)
	Good	89 (29.4)
	Fair	103 (34.0)
	Poor	61 (20.1)
**Psychological outcomes, mean (SD)**		
	Depression (PHQ-8^a^)	9.41 (5.79)
	Anxiety (GAD-2^b^)	2.71 (1.87)
**Impact of COVID-19 (1=completely disagree, 5=completely agree), mean (SD)**		
	I feel lonelier	3.55 (1.17)
	I am running into financial issues	3.20 (1.36)
	I feel more stressed	4.07 (0.97)
	I feel more anxious	3.89 (1.09)

^a^PHQ-8: 8-item Patient Health Questionnaire.

*^b^*GAD-2: 2-item Generalized Anxiety Disorder.

Table 2 displays the raw scores and the distributions of change in depression (PHQ-8) and anxiety (GAD-2) scores at 60 days from baseline for respondents who completed the baseline and exit surveys, respectively. The data in Table 2 indicate that average PHQ-8 and GAD-2 scores decreased significantly from baseline to the end of the study, suggesting improvements in depression and anxiety symptoms. There was a reduction in the mean PHQ-8 and GAD-2 scores of 18.5% and 17.2%, respectively, at 60 days compared to the baseline scores. The normality assumption of each score change is valid.

To evaluate the generalizability of our data in terms of anxiety and depression prevalence and symptoms in our baseline samples, we compared the clinical parameters between participants who only responded to the baseline survey versus those who responded to both the baseline and 60-day surveys (Tables 3 and 4). Likely major depressive disorder and likely GAD were assessed using cutoff scores of ≥10 on the PHQ-8 and ≥3 on the GAD-2, respectively. There was no significant difference (for all, *P*>.05) in clinical parameters between people who only responded to the baseline survey and those who responded to the baseline and 60-day assessment. This suggests that the mental health burden was similar between our study sample and individuals who did not complete the 60-day survey.

**Table 2:** Changes in the 8-item Patient Health Questionnaire and 2-item Generalized Anxiety Disorder scale scores for individuals who completed both the baseline and 60-day assessment (n=193).

Measure	Scores					*P* value
	Baseline score, mean (SD)	60-day score, mean (SD)	Change from baseline, %	Mean difference (95% CI)		
8-item Patient Health Questionnaire	9.30 (5.70)	7.58 (5.27)	18.50	–1.72 (–2.35 to –1.09)	<.001	
2-item Generalized Anxiety Disorder scale	2.80 (1.89)	2.32 (1.83)	17.20	–0.48 (–0.71 to –0.25)	<.001	

**Table 3:** Comparison of the prevalence rates of the risk for generalized anxiety disorder and likely major depressive disorder between subscribers who only completed the baseline survey and those who completed both the baseline and 60-day surveys.

Condition	Prevalence at baseline, n/total responses (%)			Chi-square (*df*)		*P* value
	Subscribers who completed the baseline assessment but not the 60-day assessment (n=303)	Subscribers who completed both the baseline and 60-day assessments (n=193)				
Likely major depressive disorder (8-item Patient Health Questionnaire score ≥ 10)	137/303 (45.21)	89/193 (46.11)	0.039 (1)		.84	
At risk for generalized anxiety disorder (2-item Generalized Anxiety Disorder scale score ≥ 3)	135/303 (44.55)	88/193 (45.60)	0.052 (1)		.82	

**Table 4:** Comparison of the mean scores on the 2-item Generalized Anxiety Disorder scale and the 8-item Patient Health Questionnaire between participants who only completed the baseline survey and subscribers who completed both the baseline and 60-day surveys.

Scale	Score at baseline, mean (SD)		Independent samples *t* test (*df*)	*P* value
	Subscribers who completed the baseline assessment but not the 60-day assessment (n=303)	Subscribers who completed both the baseline and 60-day assessments (n=193)		
8-item Patient Health Questionnaire	9.41 (5.79)	9.30 (5.70)	0.208 (414)	0.84
2-item Generalized Anxiety Disorder scale	2.71 (1.87)	2.80 (1.89)	0.520 (406)	0.60

To determine whether the changes in PHQ-8 and GAD-2 scores remain constant after accounting for participants’ engagement in the intervention, we used a simple linear regression model (Table 5) with participants’ fraction of mood messages answered (ie, response rates) as the main predictor. The outcome was the change in GAD-2 and PHQ-8 scores at 60 days from the baseline; thus, a negative coefficient indicates a greater improvement (larger decrease in scores) in anxiety and depressive symptoms than the reference group. We found that even when accounting for engagement in StayWell, the average improvements in both PHQ-8 and GAD-2 scores remain significant. The average improvements in PHQ-8 and GAD-2 scores controlling for engagement are –2.7 points (*P*=.001) and –0.78 points (*P*=.01), respectively.

To assess the influence of other factors (COVID-19 infection rates, self-rated health, and other demographic variables) on GAD-2 and PHQ-8 score improvements, we conducted a post hoc exploratory analysis (Table 6). This analysis also adjusts for response rates. Eight individuals lacked a valid zip code and were excluded from the analysis. Compared to females, the change in PHQ-8 score at 60 days from baseline was 2.4 points larger (*P*=.01) among males, adjusting for all other covariates; this suggests that males experienced relatively lesser improvement in depression symptoms. Having very good self-rated health was associated with less improvement in anxiety symptoms (increase of 0.83 in the GAD-2 score, *P*=.04) at 60 days from baseline compared to those with poor health and adjusting for covariates.

As a sensitivity analysis, we conducted 2 separate 1-way analysis of variance to assess the differences in the average change in PHQ-8 scores between genders and explore the association between self-rated health and the average change in GAD-2 scores. Our results show that gender had a significant effect on the change in PHQ-8 scores at 60 days from baseline (*F*_2,190_=4.106; *P*=.02). This suggests that there are true differences in the average improvement in PHQ-8 scores among male- and female-identifying participants. However, the mean change in GAD-2 scores did not differ between self-rated health categories (*F*_3,189_=2.954; *P*=.37). Thus, we cannot conclude that there are differences in the average improvement in GAD-2 scores by self-rated health.

The results in Table 2 show that both the depression and anxiety scores decreased over the 60-day period. Table 5 shows that the changes in both scores remained significant, adjusting for the response rate, as determined using a simple linear regression model (PHQ-8, *P*=.01 and GAD-2, *P*=.50). We also found that participants’ response rates are not significant predictors of improved PHQ-8 and GAD-2 scores (Table 5 and Table 6). In Table 6, we adjusted for the response rate and other demographic and clinical variables using a multivariable linear regression model, and we observed that the change in depression scores remained significant, but the change in anxiety score was not significant. However, we had previously modeled the change in PHQ-8 and GAD-2 scores by only adjusting for demographic and clinical variables, and the change in GAD-2 scores was also not significant (Multimedia Appendix 1). Therefore, the nonsignificant results for the change in GAD-2 scores are not necessarily attributed to the response rates.

**Table 5:** Simple linear regression model: responding predictor of the changes in the 8-item Patient Health Questionnaire and 2-item Generalized Anxiety Disorder scores at the 60-day exit from baseline for participants who completed both surveys.

Change in scores	Intercept, coefficient (95% CI)	Response rate, % (95% CI)	Observations, n	Adjusted *R*^2^	SE (*df*)	*F* test (*df*)
8-item Patient Health Questionnaire	–2.7 (–4.3 to –1.1)^a^	0.01 (–0.01 to 0.04)^b^	193	0.003	4.42 (191)	1.66 (1,191)
2-item Generalized Anxiety Disorder Scale	–0.78 (–1.40 to –0.16)^c^	0.00 (0.00 to 0.01)^d^	193	0.0002	1.67 (191)	1.03 (1,191)

^a^*P*=.001.

^b^*P*=.20.

^c^*P*=.01.

^d^*P*=.30.

**Table 6:** Multivariable linear regression models: demographic, clinical, and engagement predictors of the changes in score in the 8-item Patient Health Questionnaire and 2-item Generalized Anxiety Disorder scale at the 60-day exit from baseline for participants who completed both surveys.

Characteristics		Change in the 8-item Patient Health Questionnaire score^a^			Change in the 2-item Generalized Anxiety Disorder scale score^b^	
		Coefficient (95% CI)	*P* value	Coefficient (95% CI)		*P* value
Intercept		–4.84 (–8.4 to –1.3)	.01	–0.46 (–1.8 to 0.87)		.50
Weekly COVID-19 case rates per 100,000 population		–0.01 (–0.04 to 0.02)	.60	0.00 (–0.01 to 0.01)		.80
**Education**						
	At least high school	—^c^	—	—		—
	Some college	0.68 (–1.60 to 2.90)	.60	–0.62 (–1.5 to 0.23)		.20
	College	1.60 (–0.65 to 3.80)	.20	–0.63 (–1.5 to 0.22)		.15
	Graduate school	1.20 (–1.3 to 3.70)	.30	–0.93 (–1.9 to 0.02)		.06
**Self-rated health**						
	Poor/fair	—	—	—		—
	Good	1.30 (–0.92 to 3.50)	.30	0.69 (–0.15 to 1.50)		.11
	Very good	2.00 (–0.11 to 4.10)	.06	0.83 (0.04 to 1.60)		.04
	Excellent	1.40 (–1.10 to 3.90)	.30	0.42 (–0.52 to 1.40)		.40
Age		–0.01 (–0.07 to 0.05)	.80	–0.02 (–0.04 to 0.01)		.20
**Gender**						
	Female	—	—	—		—
	Male	2.40 (0.52 to 4.20)	.01	0.45 (–0.24 to 1.10)		.20
	Other	–2.80 (–8.00 to 2.40)	.30	0.08 (–1.90 to 2.00)		>.90
**Employment**						
	Full-time	—	—	—		—
	Part-time	0.44 (–1.40 to 2.30)	.60	0.45 (–0.25 to 1.20)		.20
	Unemployed	–0.44 (–2.40 to 1.50)	.70	0.13 (–0.60 to 0.86)		.70
	Other	0.00 (–1.90 to 1.90)	>.90	–0.18 (–0.89 to 0.54)		.60
**Language**						
	English	—	—	—		—
	Spanish	1.00 (–1.30 to 3.30)	.40	0.63 (–0.23 to 1.50)		.15

^a^Response rate=0.01% (185 observations); adjusted *R*^2^=0.02; SE 4.41 (*df*=169); *F*_15,169_=1.24.

^b^Response rate=0.00% (185 observations); adjusted *R*^2^=0.03; SE 1.66 (*df*=169); *F*_15,169_=1.36.

^c^—: not determined.

### Engagement With the Text Messages

Participants answered the mood text messages on average 60.0% of the time (ranging from 0% to 100%). Furthermore, 21 people did not respond to any mood-related messages, and 13 participants opted out of text messaging by texting “STOP” to our system. The 303 baseline participants were in the study for a range of 2 to 72 days (mean 59 days). Seventy participants went beyond the 60-day time frame owing to a system glitch; these participants were in the study for an average number of 63 days.

## Discussion

### Principal Findings

Participants who received the StayWell text messaging program showed improved depression and anxiety symptoms at completion of the program (60 days) on average. These results are similar to those of previous studies utilizing text messaging as a public mental health intervention to counteract the deleterious emotional and mental health effects of the COVID-19 pandemic in Canada [12-15]. Additionally, engagement with our texting study (2 messages per day) was relatively high. Response rates averaged to 60% in the daily mood check-in, and only 4% of participants opted out of the text messages during the study. This study supports the use of text messaging as a broad-based tool for improving mental health, especially in the context of a global pandemic when in-person behavioral health visits are inaccessible.

It was important to assess whether other factors (local COVID-19 infection rates, self-rated health, and other demographic variables) influenced the positive outcomes; however, we found that improvements in GAD-2 scores were not related to other measured variables. We found a greater improvement in PHQ-8 scores for female-identifying participants, but improvements held despite local infection rates or other demographic factors. These findings suggest that women may experience greater benefits in their depression symptoms from participating in the StayWell program. Nonetheless, it is particularly notable that weekly local infection rates were not related to changes in outcomes. For example, it is possible that decreases in symptoms could be influenced by reductions in local infection rates and accompanying lowered concerns of infection or reduction in policies such as stay-at-home orders. However, improvements in PHQ-8 scores persisted after accounting for changes in weekly infection rates and other covariates, suggesting that the intervention improved symptoms beyond the influence of any change in the severity of the COVID-19 pandemic in participants’ area of residence.

Over a third (36.3%) of participants did not complete the final assessment, and we took steps to assess how noncompletion impacted our results. First, we assessed whether reductions in depression and anxiety symptoms remained after controlling for participants’ response rates, and our findings suggest that the intervention may be beneficial overall even if participants do not respond. It is possible that reading messages can be beneficial even if participants do not respond to subsequent mood ratings. On average, participants who completed the study had a significantly higher response rate to mood rating messages (70%, 42/60 responses) than those who dropped out (35%, 21/60 responses) (*P*<.001). Despite no significant differences in baseline mood ratings, PHQ-8, or GAD-2 scores between completers and noncompleters, those who completed the final assessment had increased daily mood ratings (0.0037; *P*<.001), whereas those who did not complete the final assessment reported decreases in mood ratings (difference of 0.0041 from 0.0078 to 0.0037; *P*<.001). Therefore, it is possible that people with worsening symptoms respond less often or that less engagement is related with worse outcomes. It is possible that the study outcomes are biased on the basis of the mood rating post hoc analyses (attrition bias); however, this is in contrast with significant differences in outcomes remaining after controlling for the response rate. Ideally, we would be able to assess any differences in primary outcomes (PHQ-8 and GAD-2 scores), but we do not have those data for participants who dropped out of the study. This provides a mixed picture but suggests that it is more likely that participants experiencing worsening symptoms drop out of the study more often.

As stated in the trial protocol, an additional aim of this study was to test whether a reinforcement learning algorithm could improve personalization and outcomes beyond randomly selecting messages within different categories (behavioral activation and coping skills). Considering technical difficulties, we could not randomize participants into the distinct conditions, and all participants received the same intervention (random message condition). To prevent technical errors, future studies using RL should incorporate pilot work before commencing the trial to check for errors; however, we were unable to do so because we needed a quick roll-out during the pandemic. Further studies are needed to compare the effectiveness of personalizing messages using a reinforcement learning algorithm to that of a random message condition. Other studies in progress may be better suited to answer questions related to the impact of reinforcement learning models’ utility for improving personalization [23].

### Strengths

This study had a racially/ethnically diverse sample in the United States, which included 11.3% of Spanish speakers. On average, participants entered the study with mild/moderate symptoms of depression, which improved to mild symptoms at the end of the intervention period. While reductions in PHQ-8 and GAD-2 were not large, the low-intensity intervention approach is highly scalable and can accommodate a large number of people. This study also shows that participants are open to receiving 2 messages per day, including one mood response, while maintaining a high response rate (60%). Lastly, the longitudinal data collected provides opportunities for further inquiry into daily mood ratings and response rates.

### Limitations

The main limitation of this study is the lack of a control group with no or an inactive intervention. Given the impacts of the COVID-19 pandemic on mental health, the study team felt that it was unethical to withhold mental health support at this time. In addition, we were interested in assessing whether the intervention might be improved by applying a reinforcement learning algorithm to personalize messages, but technical errors prevented us from examining this. Lastly, a significant portion of the sample did not complete the final assessment, which may result in attrition bias, and further exploration of the engagement-outcome relationship is merited.

### Implications for Future Studies

Our study provides support for low-intensity text messaging interventions to improve mental health at the population level. Our data show the feasibility of sending 2 messages a day and asking for daily mood responses. Texting and other mobile interventions could serve as ways to identify individuals in need of more intensive intervention on the basis of reported daily mood ratings. Future studies should continue to assess the impacts of text messaging and related mobile health interventions for mental health and continue to assess methods including machine learning to improve personalization.

### Conclusions

Participation in a CBT-based text messaging program focused on COVID-19 cognitive flexibility, and behavioral activation and acceptance were related to improved depression and anxiety symptoms. In addition to improvements in outcomes, this study reported high levels of engagement during a 60-day intervention that sent 2 messages per day. Text messaging interventions could serve as an important public health tool to disseminate strategies for managing mental health.

## Appendix

Multimedia Appendix 1 Multivariable linear regression modeling the change in PHQ-8 and GAD-2 scores at 60-day exit from baseline for participants who completed both surveys.

## Abbreviations

BA: behavioral activation
CBT: cognitive behavioral therapy
CONSORT: Consolidated Standards of Reporting Trials
GAD-2: 2-item Generalized Anxiety Disorder scale
PHQ-8: 8-item Patient Health Questionnaire
RL: Reinforcement Learning
UC: University of California
UR: Uniform Random
