# Supplementary material for: A Text Messaging Intervention (StayWell at Home) to Counteract Depression and Anxiety During COVID-19 Social Distancing: Pre-Post Study
Source: JMIR Ment Health. 2021 Nov 1;8(11):e25298. doi: 10.2196/25298 (PMC8562416; doi:10.2196/25298)
Supplement: Multimedia Appendix 1 [file mental_v8i11e25298_app1.docx]

**Multimedia Appendix**

| Table 5. Multivariable linear regression modeling the change in PHQ-8 and GAD-2 scores at 60-day exit from baseline for participants who completed both surveys | | | | | | |
| --- | --- | --- | --- | --- | --- | --- |
| Characteristic | Change in PHQ-8*^a^* | | | Change in GAD-2*^b^* | | |
|  | Coefficient | 95% CI*^1^* | *P* value | Coefficient | 95% CI*^1^* | *P* value |
| **Weekly COVID-19 Case Rates by 100,000** | -0.01 | -0.04, 0.02 | 0.6 | 0.00 | -0.01, 0.01 | 0.88 |
| **Education** |  |  |  |  |  |  |
| At least HS | — | — |  | — | — |  |
| Some College | 0.64 | -1.6, 2.9 | 0.6 | -0.64 | -1.5, 0.21 | 0.14 |
| College | 1.70 | -0.59, 3.9 | 0.15 | -0.60 | -1.4, 0.25 | 0.2 |
| Graduate Degree | 1.20 | -1.3, 3.7 | 0.4 | -1.00 | -1.9, 0.00 | 0.05 |
| **Self-rated health** |  |  |  |  |  |  |
| Poor/Fair | — | — |  | — | — |  |
| Good | 1.40 | -0.83, 3.6 | 0.2 | 0.72 | -0.11, 1.6 | 0.088 |
| Very Good | 2.10 | -0.03, 4.2 | 0.053 | 0.87 | 0.08, 1.7 | 0.032 |
| Excellent | 1.50 | -1.0, 3.9 | 0.3 | 0.45 | -0.49, 1.4 | 0.4 |
| **Age (years)** | -0.01 | -0.07, 0.06 | 0.9 | -0.01 | -0.04, 0.01 | 0.2 |
| **Gender** |  |  |  |  |  |  |
| Female | — | — |  | — | — |  |
| Male | 2.40 | 0.55, 4.2 | 0.01 | 0.47 | -0.23, 1.2 | 0.2 |
| Other | -2.60 | -7.8, 2.6 | 0.3 | 0.17 | -1.8, 2.1 | 0.9 |
| **Employment** |  |  |  |  |  |  |
| Full Time | — | — |  | — | — |  |
| Part Time | 0.46 | -1.4, 2.3 | 0.6 | 0.46 | -0.24, 1.2 | 0.2 |
| Unemployed | -0.49 | -2.4, 1.4 | 0.6 | 0.10 | -0.62, 0.83 | 0.8 |
| Other | -0.05 | -1.9, 1.8 | >0.9 | -0.20 | -0.91, 0.52 | 0.6 |
| **Language** |  |  |  |  |  |  |
| English | — | — |  | — | — |  |
| Spanish | 1.00 | -1.3, 3.3 | 0.4 | 0.63 | -0.23, 1.5 | 0.2 |
| Constant | -4.38 | -7.69, -1.07 | 0.01 | -0.25 | -1.50,1.00 | 0.7 |
| Observations | 185 |  |  | 185 |  |  |
| Adjusted R2 | 0.02 |  |  | 0.03 |  |  |
| Std. Error (df = 170) | 4.41 |  |  | 1.66 |  |  |
| F Statistic (df = 14; 170) | 1.29 |  |  | 1.39 |  |  |
| *^1^* CI: Confidence Interval, *^a^* PHQ-8: Patient Health Questionnaire-8, *^b^* GAD-2: Generalized Anxiety Disorder-2 | | | | | | |
